# The optimum condition for electric vehicles’ battery powering factors to travel distance: A model-based approach

**DOI:** 10.1016/j.heliyon.2024.e39719

**Published:** 2024-10-28

**Authors:** MD Shouquat Hossain, Audrius Senulis, Laura Saltyte-Vaisiauske, Mohammad Jakir Hossain Khan

**Affiliations:** a Department of Electrical and Electronic Engineering, International University of Business Agriculture and Technology (IUBAT), Dhaka, 1230, Bangladesh; b Engineering Department, Faculty of Marine Technology and Natural Sciences, Klaipeda University, H. Manto 84, 92294, Klaipeda, Lithuania

**Keywords:** Electric vehicles, Batteries, Integrated model, Modeling and validation, Power sources performance

## Abstract

The development of electric vehicles (EVs) and their power source systems (PSS) is a rapidly growing field of technology. However, the EV's travel distance (range between charging stations) depends on the agility of the PSS, or battery capacity system. EV driving range and battery capacity are the two most significant technical challenges in commercializing EVs. This study aims to propose an integrated model that identifies the optimal energy factor orientation, enabling EVs to cover the maximum travel distance and reach the charging station for their next trip. Additionally, the artificial intelligence (AI) and statistical models were integrated and applied to predict, validate, and explain how energy factors affect the driving range of EVs. The developed models and validations revealed that maintaining precise assimilation of battery power factors can vary the EV's travel distance from 60 to 610 km. In this case, we have identified 77.5 kWh battery capacity and 14.5 kW charging capacity as the optimum power source factors. After 5.5 h of charging, various adjustments to power source factors allow for optimum battery performance. We have also proposed the central composite factorial design (CCFD) to compute the impact of energy factors on travel distance. The study used the response surface methodology (RSM) and an in-house-developed AI-based algorithm to achieve the research results. The alignment percentage between model-predicted data and real-time outputs showed an extremely high precision of over 95 % and confidence in the findings' reliability.

## 1 Introduction

Globally, for more than 100 years, the popularity of EVs has been on the rise due to various advantageous and eco-friendly features, such as negligible toxic emissions and radiation, reduced dependence on fossil fuel consumption, improved productivity, lower noise, and so on [1,2]. The additional benefits, such as minimum noise pollution, low maintenance costs, controlled CO_2_ emissions, optimum utilization of vehicle space, and user-friendly operations, have positioned EVs as the most appealing means of transportation. Additionally, EVs may be a future energy-sharing technology for the grid (vehicle to grid) [[3], [4], [5]], but the traditional grid system might impact the adoption of such technologies [6]. However, continuously upgrading an integrated EV power system is a highly demanding and fast-growing research trend. When the charging technique (slow, fast, rapid, or ultrafast) affects the vehicle's traveling range, the scarcity of charging sources and related technological parameters present significant engineering challenges. Hence, the mentioned technical concerns push manufacturers to pay extra attention to developing an optimal and easily adaptable model for upgrading an EV's inclusive PSS.

Very few research initiatives have focused on the mentioned area. For instance, Naumanen et al. [7] conducted a comparative survey on heavy-duty EV battery development initiatives in the USA, EU, China, and Japan. They aimed to analyze and develop the most technically suitable batteries for EVs in the above regions. However, the authors concluded that technical concerns, state regulations in battery recycling, and environmental effects limited future developments. In another study, Lokesh and Min [8] devised a structure for an EV charging power station in Singapore. Their research reveals real-time information about the distances between EVs and charging stations. They used Java and Google Maps to guide charging times and other locations.

EV range is essential and may influence user opinion. Back et al. [9] proposed a general method to estimate and enhance the range of an electric vehicle by varying the accuracy and complexity of the models describing the route, vehicle, and battery. Krause et al. [10] performed a simple statistical study on EV users' viewpoints based on rapid-charging technology in Germany. The author asserted that a novel charging technology can recharge the battery to 80 % in 20–30 min, depending on the vehicle's model. However, rapid charging technology could influence a trip. To test the effectiveness of the machine learning (ML) approach, Sun et al. [11] proposed a machine-learning approach for predicting the tentative travel distance of battery electric vehicles (BEVs). The author utilized a gradient-boosting decision tree algorithm to address the relationship between battery productivity and ‘range anxiety’ on the BEVs to the driving range. In a related study, Pan et al. [12] modeled the travel distance of EVs by considering driving conditions using the Markov algorithm and the Kernel Principal Component approach. The author applied this method to predict the impacts of acceleration range, traffic conditions, charge energy consumption, and other traffic dynamics on the driving range.

Besides, Alamin et al. [13] and their team present a battery digital twin structure by properly reflecting run-time battery dynamics. The digital twin continuously runs a state of health (SoH) model to estimate the maximum loss of battery capacity and constantly retrains a state of charge (SoC) model to estimate battery aging, which includes more accurate non-linear behavior. Implementing battery-aware charging strategies that control charge cycles more than discharge cycles can reduce battery age. Because average SoC plays a significant role in battery aging, these algorithms minimize standby time between the end of the charge and the EV's unplugging. Naeem et al. [14] presented a comprehensive approach to optimize eco-driving, aiming to minimize energy consumption and extend battery life for electric vehicles. They collaboratively identify the issue by considering various conflicting constraints, such as traffic signals, preceding vehicles, speed and acceleration limitations, input torque checks, and limits on the battery's SoC and charging/discharging rates. Chen et al. [15] tested machine learning-based predictions. They used a public dataset of residential EV charging points to show that a simple tree-based ML model trained on user behavior can reduce forecasting errors by up to four times compared to older models. Wenig et al. [16] proposed a guideline to understand the powering source versus infrastructure effects on plug-in hybrid EVs. The author's investigation revealed that the frequently installed charging facilities may be beneficial only for long-range traveling vehicles. In a related study, Zhou et al. [17] investigated the optimal battery EV range and discovered charging behaviors in the all-electric driving range. The authors raised three essential points to determine the optimal battery range, such as (a) passenger travel demand, (b) charging infrastructure, and (c) individual charging behavior.

On the other hand, Das et al. [18] conducted a technological review of EV charging standards, infrastructure, and their effect on grid integration. The author also proposed a conceptual model for calculating the so-called societal cost, which covers fuel, electric vehicles, and battery degradation costs in the energy management problem. The same research group also examined the size of batteries and the energy supply system's capacity for charging. The authors ignored the effects of the battery charging pattern and the non-linear dynamics of techno-economic factors [19,20]. Additionally, the authors adopted the ‘Deep Neural Network (DNN)’ and the ‘Squirrel Search with Improved Food Storage (SS-IFS)’ techniques to understand the stability and safety of the EV's battery system [21]. The study also suggested a model for optimizing EV charging schedules using municipal charging stations with fast charging utilities. The investigation acknowledged the importance of further research on model development to identify the optimum factors for transformer upgrades [22].

Despite the above developments, many factors still make predicting EVs' future utilization and spread challenging. Among these considerations are the battery's capacity, the EV's charging time, the proximity of charging stations, the EV's travel distance, the battery's performance, driving weather conditions, driver actions, and the impact of the road landscape. If EV drivers hang around indifferent about ‘when and where’ to charge their EVs, they may find themselves perplexed, even though they maintain a reasonable charge level. This is due to the smaller margin of error in the fuel/charge indicator compared to internal combustion engine (ICE) cars [8]. To address this issue, this paper proposes a statistical model of EV battery powering factors with respect to travel distance that is suitable for EV charging at the optimum conditions. More specifically, we integrated the model to identify the optimal energy factor orientation, which allows EVs to cover the maximum travel distance and arrive at the charging station for their next trip. In this manner, we incorporate the analysis of different EVs and their battery power, the capacity of travel distance, and the overall analysis methodology to build a statistical model and extract the battery range predictions.

The current work employs a numerical method known as “analysis of variance” (ANOVA) and AI concepts, which can effectively address research gaps by identifying numerous complex energy factors. As a result, it is highly desirable to develop a wide-range multivariable EV model that considers the adjustable battery charging capacity, required charging time, and distance between charging stations. In this research, we used various global EV brands and models, as well as Google Maps, to estimate the range of EVs and the distance between charging stations. It is essential to know that the EV battery is the primary energy source, so choosing the appropriate battery for BEVs is critical for achieving maximum driving ranges. In EVs, the battery size, weight, and price are significant issues for public transport vehicles (i.e., buses, etc.) and private cars. A small battery generally requires more frequent charging but does not have the option to complete long trips. Moreover, a larger battery size necessitates more time for charging and increases the vehicle's weight, thereby escalating energy consumption, rendering it significantly inconvenient to use for extended journeys.

However, this is the first study of its kind to propose an integrated model that uses both polynomial statistics and AI ideas. The main goal is to find the best factors (charging time (A), the amount of electricity needed to charge the battery (B), and the capacity of the EV battery (C)) for figuring out the shortest and longest driving distances that EVs can go. We used the ANOVA method to investigate the impact of key components of the EV's power system (PSS), such as the battery, on the travel distance and the interplay between these factors. Here, we have utilized the RSM, a functional statistical method, to determine the optimal integration of battery dynamics. This method can be highly flexible in guiding the design of EV powering systems, ranging from research to commercial scale. Another significant statistical tool, ‘design of the experiment (DOE),’ is a highly recommended scientific approach for conducting model-based precise prediction and experimental validation research [[23], [24], [25], [26]].

This means that the polynomial statistical models created using the above methods form a broad framework for studying the energy aspects of EVs, specifically battery power dynamics. This one-of-a-kind mixed AI-statistical model will include essential factors for finding the most extended electric vehicle (EV) travel distance at the best battery-delivered energy variable arrangements, offering a promising future for EV energy optimization. We utilized a neuro-fuzzy hybrid system and a polynomial model to determine the optimal arrangement of variables (A, B, and C), explore the relationships between the independent variables, and compare the model's predictions with the response, Y (travel distance). Hence, it will be more advantageous to forecast the outcome before the investigation and decision-making, saving valuable time and money.

## 2 Background and state of the art

**Fig. 1:**
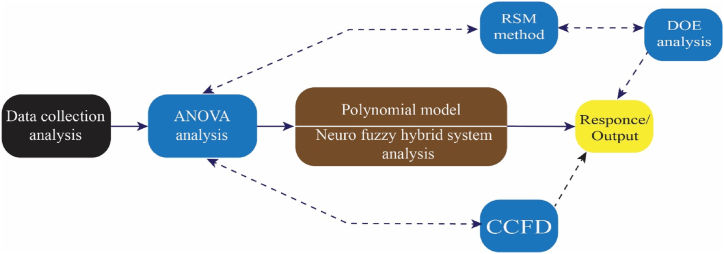
Overview of the analytical process.

This research has proposed and implemented two numerical approaches. The first is data analysis. This approach selects a model-free, history-data-based method for EV types, driving ranges, battery capacity, and charging levels without requiring significant computational effort. Second, we supply and analyze the data using an ANOVA model based on the RSM, DOE, and CCFD analyses. Finally, we combined and studied a polynomial model and a neuro-fuzzy hybrid system to determine the optimal variable configuration for achieving the desired response or output. The following section provides more details on model development for EV performance. Fig. 1 provides an outline of the analytical process.

Fig. 1 shows the data collection and technology selection components where pathways of establishing interface between statistical methods and specific AI techniques can been noticed.

**Fig. 2:**
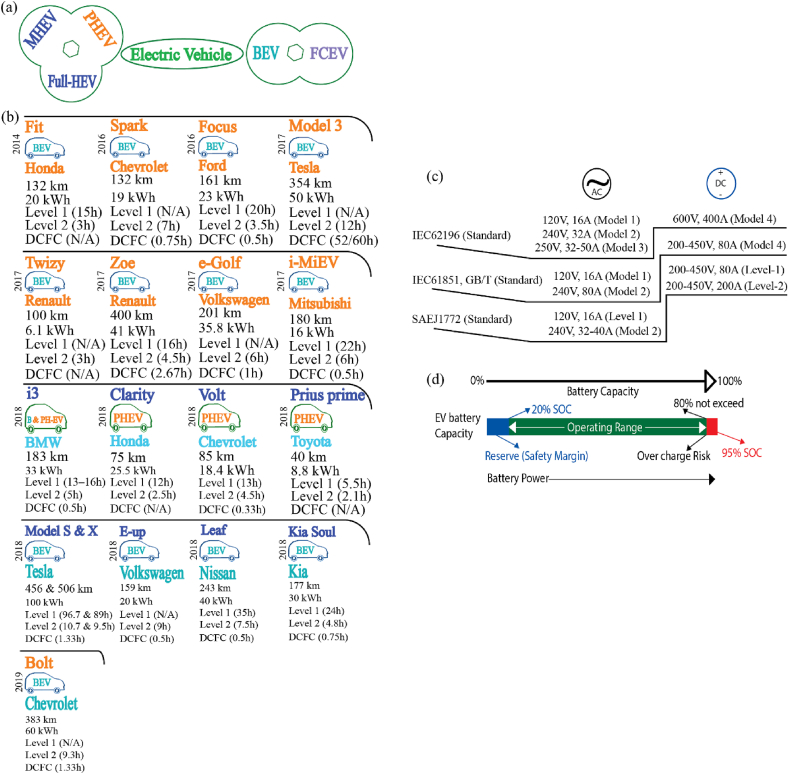
EV classification and data collection. (a) EV types; (b) EV models and their driving ranges, battery capacities, and charging levels; (c) EV charging standards; and (d) an overview of the EV battery SoC.

Fig. 2(a) shows the classification of the EV technology type. Fig. 2 illustrates the classification of EV technology into hybrid and non-hybrid all-electric types, represented as HEVs and AEVs, respectively [27,28]. We have kept BEVs under the AEV cluster. BEV relies solely on an external power supply from the grid to charge the storage unit. Additionally, we have classified fuel-cell electric vehicles (FCEVs) as AEVs. However, FCEV does not require an external electrical energy supply. On the other hand, a plug-in hybrid electric vehicle (PHEV) is a class of HEV that can use the grid for power. Fig. 2(b) shows identical information on affordable EVs manufactured by different companies [[29], [30], [31], [32], [33], [34], [35], [36], [37], [38], [39], [40], [41], [42]], which is fundamental information in this study. Most vehicle manufacturers are currently developing BEVs. The data includes battery capacity, range, and charging methods.

An appropriate EV charging station is critical to charging EVs at the expected level. EVs can charge with several charging standards, as shown in Fig. 2(c). Europe generally uses the International Electrotechnical Commission (IEC) charging standard, while the United States uses the IEEE standard. China utilizes the GB/T National Standard, which shares similarities with the IEC's principles. However, several factors control the charging standards, including the power level (kW), electricity usage, and battery type. For example, a moderate charging station power supply is about 3.3 kW, and the fast DC charging power could be about 50 kW [43]. However, it is required to charge the battery from the single-phase power supply or an AC-powered level 2 charging or to use an integral compact EV, such as a plug-in hybrid, which might help get the most efficient charging level. For example, in Europe, users can use electricity from the conventional AC single-phase (230V) power supply via external vehicle accessories and then convert AC to DC for charging the battery. These facilities are available in low-voltage places. Otherwise, more EV users will demand fast DC charging up to about 50 kW, which could overload the grid [43]. As the demand for EV charging stations continues to grow, the study covered some necessary equations that could be useful to calculate EV battery charging status, travel range, charging duration, etc.

On the other hand, Fig. 2(d) shows the battery capacity and SoC. This is mainly to determine the safety margin and overcharge risk. Appendix A (Supplementary Section 1) contains the EV battery charging equation information.

## 3 Proposed methodology and the models

### 3.1 Model development for identification of energy factors related to EV performance

Charging time, electric power, and battery capacity were looked at as separate factors to figure out and explain how each factor affected the estimated range that EVs could travel (the specific equation can be found in Appendix A, Supplementary Section 1). However, according to the analysis process, the model works based on input and output data, which has a complex structure and multiple layers that make it difficult to understand. Therefore, we have designed an adaptive neural fuzzy inference system (ANFIS) to observe parameter modification and decision-making based on input-output training information. Appendix A (Supplementary Section 2) contains more information.

The term ‘Response’ in this model refers to the travel distance achievable by EVs. In subsequent equations, the ‘Response’ will be written as ‘Y.’ The charging time, charging capacity, and battery capacity are considered independent variables and will be termed factors A, B, and C, respectively. The RSM is a statistical technique widely used to quantify the relationships between the response (R) and the identified input factors [44,45]. Model development has adopted the CCFD sub-section under the RSM. The CCFD algorithms are made to figure out the coefficients of a proposed model. The values are usually normalized between −1 and 1, each parameter's highest and lowest ranges. This helps to create an experimental design based on the conditions being studied. The required number of trial runs was computed by solving equation (1), which has been summed to 16, as presented in Table 1. The data analysis was conducted randomly to reduce the unplanned variables. The number of runs (arrangements of the parameters) in the calculation depends on the value of k; the constant p stands for the values 0 for k < 5 and 1 for k > 5; and the number of central points, n_c_. Analyses of the central points will be carried out in triplicate to obtain average values:

(1) $$n=2^{k-p}+2*k+n_{c}$$

where *2*^*k-p*^ is the points of a factorial design, *2∗k* is the axial points, and *n*_*c*_ is the central points.

**Table 1:** Primary coded level for experimental design.

Charging time (A) hr	Charging capacity (B) kWh	EV battery capacity (C) kWh	**Travel Distance (Y) km**
8.00	18.00	10.00	80
8.00	3.00	100.00	495
3.00	3.00	10.00	65
5.50	18.00	75.00	580
8.00	18.00	100.00	610
5.50	3.00	75.00	498
3.00	3.00	100.00	490
3.00	18.00	10.00	70
5.50	10.50	75.00	561
5.50	10.50	75.00	560
3.00	10.50	75.00	556
5.50	10.50	10.00	60
5.50	10.50	100.00	575
8.00	10.50	75.00	565
3.00	18.00	100.00	601
8.00	3.00	10.00	70

All 16 investigations (runs) employed random sampling using the RSM method, with k = 3 and n = 2. We carefully examined the data for factors A, B, and C using the design formulas. The developed RSM method suggested an idealistic experimental design.

The factor values will be able to identify the optimization of the real objective formula after the initial stage of adjusting the polynomial function with the statistical data. This optimization process takes place during the material time. The coefficient can assess the alignment between the model-predicted data and the collected data for the polynomial model values *R*^*2*^ and *R*^*2*^_*adj*_, as expressed in the equation form [[46], [47], [48], [49], [50]]:

Equations (2), (3):

(2) $$R^{2}=1-\frac{\sum S_{residual}^{2}}{\sum S_{\mathrm{mod}\;el}^{2}+\sum S_{residual}^{2}}$$

The variables *S*^*2*^_*residual*_ and *S*^*2*^_*model*_ represent the ratios between the mean square of regression and the mean square of residuals. Equation (3) allows for the adjustment of the *R*^*2*^ to account for the number of predictors in the model, which is known as adjusted *R*^*2*^_*adj*_.

(3) Radj2=1−∑Sresidual2DgFresidual(∑Smodel2+∑Sresidual2)/(DgFmodel+DgFresidual)

where *D*_*g*_ *F*_*residual*_ degree of freedom of residual and *D*_*g*_ *F*_*model*_ degree of freedom of model.

The response, Y (travel distance), of the developed model was computed by applying equation (4). For the computation of the optimal points (maximum, minimum, or saddle), it is required that the polynomial functions comprise quadratic terms as per the equation presented below:

(4) $$Y_{TD}=\beta_{{}^{\circ}}+\sum_{i=1}^{k}\beta_{i}\chi_{i}+\sum_{i=1}^{k}\beta_{ii}{\chi_{i}}^{2}+\sum_{i=1}^{k-1}\sum_{j=i+1}^{k}\beta_{ij}\chi_{i}\chi_{j}+\varepsilon$$

where *Y*_*TD*_ is the predicted response; *Y*_*TD*_ will be written as ‘Y’ in the following section. Some statistical constants and coefficients exist in equation (4); for instance, the number of considered variables has been represented by *k*; hence, *β*_*ο*_ and *β*_*i*_ are the constant and linear coefficient representations, respectively. As mentioned earlier, understanding the interaction intensity between parameters can be computed from this model; thus, *β*_*ii*_, and *β*_*ij*_ represent the coefficients of the battery powering factors (parameters) interaction, whereas *X*_*i*_ signifies the adjustable variables and *ε* is the residual linked with the experiments.

Regression and graphical presentations have explained model evaluation, experimental design significance, and validation logic. The analysis conditions have been assessed via the surface 3D plot and regression scrutiny of the independent variables, which vary with each dependent variable.

A p-value, which also denotes the level of interaction between other independent variables, measures the significance of the independent variable selection. A small p-value indicates the degree of significance of the associated variables. Previous publications [51,52] provide methods to determine the p-value and other related statistical parameters.

The second-order models have been tested using the ANOVA and F-value analysis methods. Because the ratio conforms to an F distribution with one numerator degree of freedom and two denominator degrees of freedom, statistical theory commonly uses the F-value method. That is why it is commonly known as the analysis of variance F-test. The following equation (5) is the ANOVA F-test:

(5) $$F=\frac{Mn_{RG}^{2}}{Mn_{RD}^{2}}$$

where *Mn*^*2*^_*RG*_ is the mean of square regression and *Mn*^*2*^_*RD*_ is the mean of square residual. The significance of the F-value in analysis during model development and the degree of freedom (DoF) in choosing the variables and their range settlement are important considerations.

Equation (6) can formulate the so-called ‘variation indicator coefficient’ for calculating the probable model error.

(6) $$\text{CV}=\frac{\text{SD}}{\text{mean}}\times 100$$

It is worth mentioning that the *CV* value can be accepted if it exceeds 10 % for any model to get a reproducible model. The *SD*, however, is the standard deviation.

### 3.2 Development of design methodology

The analysis design made by RSM led to the creation of 16 sets of computing arrangements, with each one changing the charging time (A) in hours (hr), the charging capacity (B) in kWh, and the EV battery capacity (C) in kWh. The computed "Response" (Y) (km) of the developed model was the travel distance (km) that EVs covered. Table 1 lists the computational outcomes, taking into account the design of the energy factor analysis.

## 4 Results and discussions

### 4.1 Model explanation and parameter effects analysis by the RSM algorithm

It is always desirable to understand the correlations between the variables (A, B, and C) and their impacts on the “Response” (Y). The RSM was used to determine how the variables' interactions affected the response, which way the variables should be arranged to get the highest response value and to estimate the significance of the model [49,50]. The RSM-based quadratic model for achieving the maximum Y, ‘travel distance’ (*TD*), can be calculated by solving equation (7):

(7) $$\begin{array}{c} Y_{\text{TD}}=\left(0.28\times A\right)+\left(0.002\times B\right)+\left(0.42\times C\right)+\left(0.025\times \text{AB}\right)‐\;\left(0.032\times \text{AC}\right)‐\;\left(0.030\times \text{BC}\right)\\ ‐0.55\times A^{2}+0.038\times B^{2}‐0.13\times C^{2}‐\;5.94 \end{array}$$

**Fig. 3:**
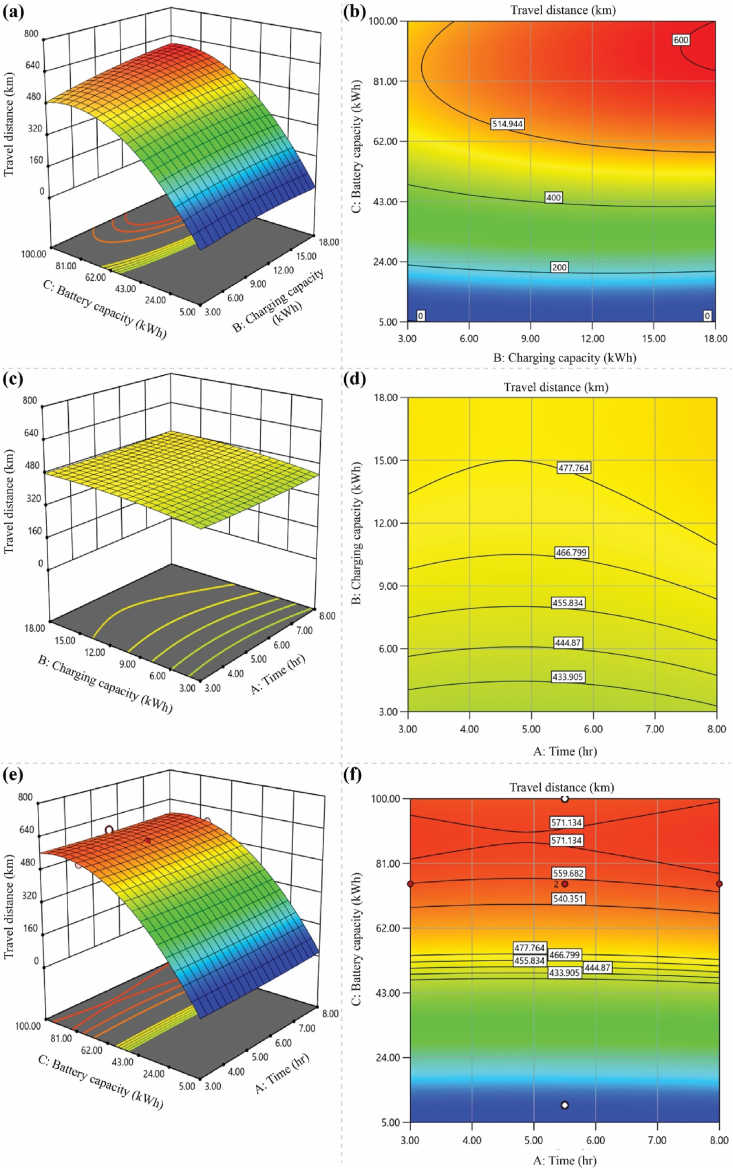
Both 3D (4a, 4c, and 4e) and 2D (4b, 4d, and 4f) contour plots indicate the optimum arrangement of two variables over the response (Y), where the blue color signifies the lowest and red the uppermost response level ranges accordingly.

Fig. 3 expresses the ‘3D surface’ and ‘2D contour’ plots for different variables. Theoretically, we can justify the interaction composition between two variables by fixing one variable constant at the central point in equation (7). The 3D plot, in combination with the contour plot, has also been used to validate the results for optimum arrangements of the variables for the highest travel distance at the contour surface. On the other hand, the 2D plots offer more detailed information about the battery powering factors' values and their impact on the response (travel distance) in various analytical stages.

Fig. 3(a)and (b) illustrate the impact of ‘battery capacity’ and ‘charging capacity’ on the travel distance (Y), assuming a static ‘charging time’ at the surface's central points. The travel distance showed an increasing trend with the development of batteries and charging capacity up to a specific level. The 3D response surface plot associated with this arrangement reveals an increasing Y (Response) value trend. Maintaining the battery capacity (C) at 77.50 kWh will lead the response (Y) point value to reach its peak within the 588 km–605 km range. When the battery capacity increases from 80 to 100 kWh, it shows the stationary-phase response value. In response, additional battery increments showed similar trends. Therefore, 77.5 kWh represents the optimum battery capacity (C). On the other hand, the 3D contour plot reveals that the charging capacity (B) exhibits greater optimum response values in the 15–18 kW range. Fig. 3(b) reveals a sharp upward trend in the travel distance (Y) at battery capacity values ranging from 77.5 to 80.0 kWh. This trend began at the charging capacity (B) of 14.5 kW, and the travel distance (Y) response values continued to persist between 581 km and 610 km. The increase in charging capacity (B) above 15 kW did not indicate any momentous improvement in the travel distance covered, which shows that the optimum zone starts at 14.5 kW charging capacity. The literature has designated charging capacity tuning as a central parameter for travel distance driving, which correlates with battery capacity, as it can alter the battery performance of various EVs [6].

The 3D and 2D response surface graphs in Fig. 3(c) and (d), on the other hand, show how “charging capacity (B)” and “charging time (A)” affect the response and travel distance (Y). These graphs show that the ‘Y’ value (km) increases as the charging capacity rises from 3.00 to 14.25 kWh. However, the optimum response value reached a stationary phase in the range of charger capacity values from 14.25 to 18.00 kW, ranging from 425 km to 580 km. Interestingly, changing the time (hr) did not significantly affect the results. Therefore, considering factors (A) and (C) as the primary parameters influencing the travel distance, we can attain a maximum travel distance of 589 km, although not the most optimal result. The charging time, among the three battery-powering factors, is not an adequate parameter. The literature also demonstrated that the battery charging time is the only factor affecting travel distance [17]. Kostopoulos et al. [3] disclosed that the charging time does not significantly affect the traveled distance when the nature of recharging (the percentage of charge remaining in the battery) remains constant at a certain level. For instance, their model-based computation showed that batteries for EVs charging for 1 h could offer traveling distances from 44 km to 71 km when the so-called SoC area is tuned from ‘80%–100 %’ to ‘20%–80 %’, respectively. This literature strongly supports the study's validation results.

Additionally, Fig. 3(e) and (f) illustrate the interaction impact of battery capacity (C) and charging time (A) on response Y. In this case, the battery capacity tuning improves sequentially and reaches optimal. Maintaining the battery capacity at 75.5 kWh allows a maximum travel distance of 580 km. In this factorial interaction, the charging time did not significantly impact the response value.

This section concludes that the interaction between battery capacity (C) and charging capacity (B) is the main factor significantly affecting travel distance. Maintaining the battery capacity at 77.5 kWh and the charging capacity at 14.5 kW can achieve the optimum conditions. A maximum travel distance of 610 km can be achieved if the battery is charged for 5.5 h by maintaining the mentioned power factor combination.

### 4.2 Model accuracy analysis

The analytical accuracy of the developed model for EV energy management systems has long been one of the foremost concerns for researchers, academics, and manufacturers. Among a few earlier works, one of the research groups proposed a general statistical model that expressed the optimum positioning of charging stations for EVs used for leisure trips but needed to explain in detail the model's accuracy and level of fitness. Furthermore, the work should have accounted for potential errors due to the omission of model variable order terms and the absence of factorial interactions. This work employs widely used statistical formulations to elucidate the significance of the developed model.

The study used the ANOVA approach's parameters in conjunction with additional suggestive statistical diagnostic tools to assess the model's fitness:

1. The model's predictions were tested for accuracy using the “range of factor arrangements” for the dependent variable.
2. The response values (Y) for the 16 arrangements are calculated, resulting in normal probability and residual plots for the travel distance, as shown in Fig. 4(a). This method checks the relationship between the predicted data and the fine-tuned variables (A, B, and C) values.

**Fig. 4:**
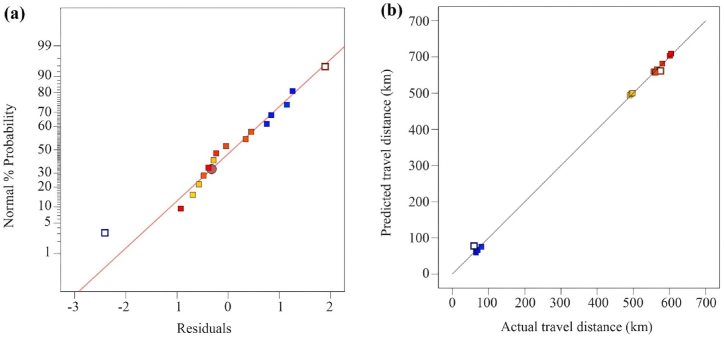
(a) Normal plot of residuals and (b) plot for predicted vs. actual travel distance in km.
3. It can provide insight into the normality of the data distribution.

A normal distribution analysis should align the outlined data closely with a visualized straight line. If the plotted data closely aligns with the linear line, we can define it as normally distributed; any divergence from the line will confirm this definition. Fig. 4(a) displays the alignment of the data and the degree of agreement with the results. We found these using the AI integrated ANOVA method, and the residuals revealed the difference in standard deviations between the model's predicted values and the database values (in this case, Google Maps values). Fig. 4(a) recommends avoiding repetitions of the obtained results, as no additional noticeable error is associated with normality.

The model strongly recommends practicing estimating data analysis errors (residuals) when developing statistical models. An accurate examination of residuals can testify to the acceptability of the data analysis methods for selection and computation. However, during the regression analysis, the errors typically develop randomly. Fig. 4(b) presents the error analysis results, which showcase the randomly scattered residual and projected value distributions. The randomly spread data points show a minimal difference between the actual and model-predicted data. The following sections and subsections will discuss the possibility of achieving a highly accurate alignment between the actual travel distance data and the model-predicted values.

**Fig. 5:**
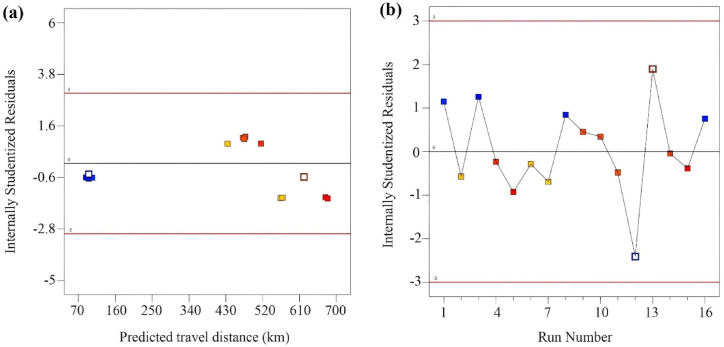
(a) residuals vs. predicted travel distance, and (b) residuals vs. number of test runs.

Determining the number of deviation points from the arranged “set of variables” is crucial for evaluating the significance of the developed experimental design or variable compositions. The ‘outlier t’ measurement is a unique tool applicable to this purpose. Fig. 5(a) and (b) illustrate the outlier t plot for travel distance on the individual arrangements of the battery variables studied. The residual values for each variable arrangement stay within the range of ±3.00. This means there is a high chance that the model fits the response surface, and there is little room for error in the data analysis. However, any variable arrangement found in the range of ±3.00 can be marked as an insignificant data analysis arrangement, necessitating further investigation or recalculation.

### 4.3 Perturbation graph

**Fig. 6:**
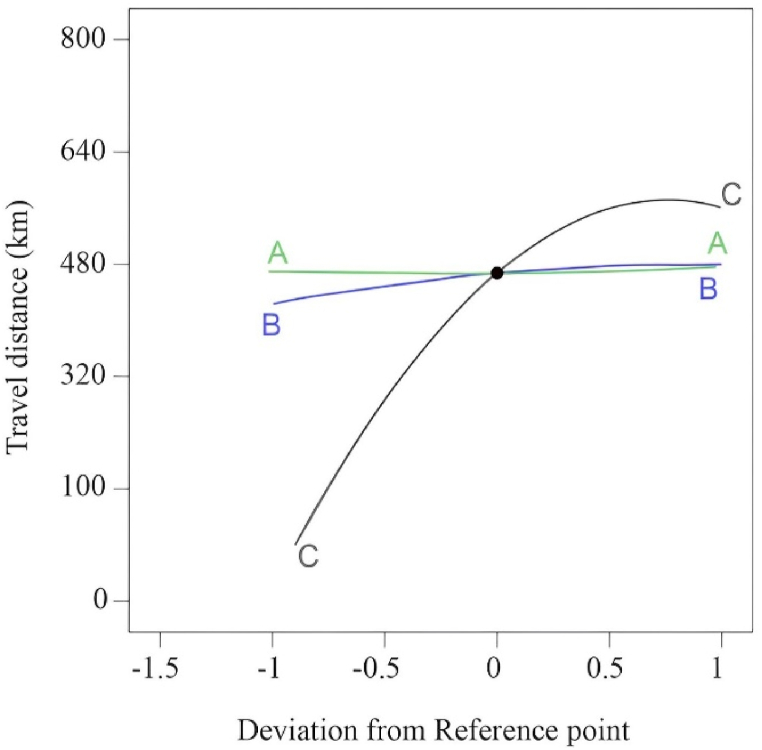
Deviation plot for perturbation parameters.

A statistical indicator known as a perturbation plot can be used to demonstrate the precise impact of each variable on the response, which is an additional crucial issue in energy factor modeling. The design plot's central point serves as its basis. Therefore, this plot makes it easier to compare the significance of each battery powering variable (A, B, and C). Fig. 6 displays the perturbation chart for the travel distance (Y) about A, B, and C. The perturbation plot shows the impact of a specific variable at specific points. Simply shifting one battery variable (A, B, or C) at a time over its optimal point while leaving the other two variables at its center allows for plotting the values of Y's (travel distance).

The model generated the perturbation plot in Fig. 6, which illustrates the impact of each independent energy variable of the battery on the travel distance. Fig. 6 shows that the changes in the dynamic trends of travel distance are susceptible to both energy supply variables for the EV systems. It can be observed that there is a sharp bend in the curves for battery capacity (C) and charging capacity (B). In comparison, the semi-flat charging time curve (A) is less sensitive to the effect of a change in travel distance, i.e., on response efficiency. In other words, the charging time does not significantly affect the EV distance traveled when comparing battery and charging capacity.

### 4.4 ANOVA appliance for model significance evaluation

The study used ANOVA to examine the individual importance of each battery factor and the strength of their interaction. The results are displayed in Table 2.

**Table 2:** Statistical parameters for sequential models.

Source	F-Value	*p*-value (Prob > F)
Model	826.80	<0.0001
A-Charging time	0.96	0.3645
B-Charging capacity	69.20	0.0002
C-Battery capacity	6416.55	<0.0001
AB	0.010	0.9229
AC	0.037	0.8535
BC	49.50	0.0004
A^2^	0.73	0.4254
B^2^	6.08	0.0487
C^2^	382.76	<0.0001

Lack of Fit: 264.97; R-Squared: 0.9992; Adj. R-Squared: 0.9980.

The model's F-value of 826.80, which indicates that only 0.01 % of calculations are error-free and that the probability of error due to noise is negligible, is a signal of its significance. Less significant Prob > F values, <0.0001 (in standard analysis, less than 0.05 % is acceptable), are a strong indicator of the importance of model factors (variables). Model variables are considered non-significant when their values exceed 0.100. According to the ANOVA, the charging capacity and battery capacity have a significant impact on the response. The Prob > F values for charging and battery capacity are 0.0002 and <0.0001, respectively, as shown in Table 2. This implies that the study's response becomes crucial when the battery capacity value fluctuates, and the charging capacity fails to maintain proper control within a predetermined range. The R^2^ is commonly used in statistical modeling to reduce response variability. However, a high R^2^ value alone does not indicate a better alignment for a regression model. We can determine it by solving equation (8), which must remain between 0.0 and 1.0. Theoretically, the R^2^ value closer to 1.0 designates an improved alignment of the model with the actual data. The value at 0.9992 to R^2^ (very close to 1) signifies the model selection and improvement for this study. The ‘R^2^_adj_’ (adjusted R^2^) is another statistical factor that indicates the number of variables considered in the analysis. It determines whether it is significant enough or requires additional adjustments to the number of factorial analyses essential for the model. In statistical analysis, the model always expects a lower value of ‘R^2^_adj_’ than ‘R^2^’, indicating that no additional terms are required to prove the model's significance. During analysis, we found the R^2^_adj_ value to be 0.9980, which is lower than the R^2^ value, indicating that no additional term is required. This time, the “Lack of Fit F-value” of 264.97 means that the value is good because there is still only a 4.66 % chance that it is wrong. This could be primarily because of the noise in adapting the AI-based data training procedure.

Primarily, the signal-to-noise ratio is used to determine adequate precision. This ratio can show the tuning range of the model's factors, which can be used to find the design level and response prediction range for the factorial analysis platform using equation (8). The standard for adequate precision should be greater than 4.0. The outcome in this case has a value of 66.11, determined using equation (8):

(8) $$\frac{\max\left(Y_{p}\right)-\min\left(Y_{p}\right)}{\sqrt{\frac{p\omega^{2}}{n}}}$$

where *Yp* are the anticipations at the arrangements design of the parameters, *ω*^*2*^ is the residual mean square. *p* is the absolute term in the model and *n* is the number of runs in the design.

This technique is highly applicable to the precision regulation of AI. With the support of RSM targeted predictions that would increase the dependability for EV companies to improve and control dependability on AI.

## 5 Application

**Fig. 7:**
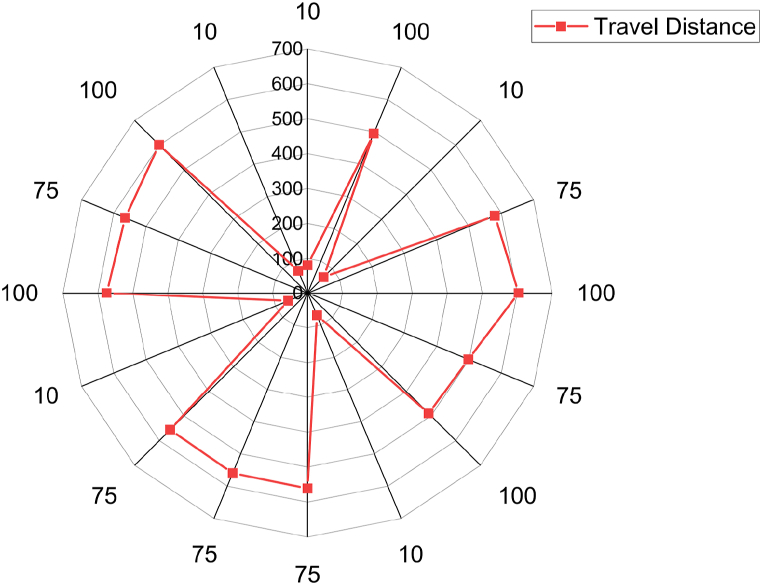
EV battery capacity and travel distance spider map.

In order to find the most adaptable response (travel distance), the technical features of the battery in terms of size and energy storage dimensions are essential. Therefore, the research findings will significantly affect installing charging stations within the recommended distance range. In this scenario, EVs will encounter no significant issues while driving maximum travel distances. This study analysis reveals that an EV with a minimum battery capacity of 10 kWh can cover a distance of 60–80 km (km). A 100-kWh-capacity battery-based EV can travel up to 610 km for long distances, as shown in Table 1 and Fig. 7. However, the result also suggests that maintaining the battery capacity at its maximum level (77.50–100 kWh) will enable the EV to reach its optimum range of approximately 588–610 km. The model also identified that the optimum charging capacity is between 15 and 18 kW. Therefore, the EV charging station distance can be maintained at an average of 60 km because it can cover all types of BEVs. For example, the average distance between charging stations in China is approximately 50 km [53]. The European Union recommends an average distance of 60 km (37 miles) between European charging stations. However, those countries’ areas are large and need to install more swift and slow chargers [54].

According to the International Energy Agency (IEA), China planned to install 680000 EV charging stations with slow chargers in 2021. However, growth was slower during the pandemic than in 2019. In China, many places still need EV charging stations because they rely on fossil fuels. Some big cities, such as Beijing, Shanghai, Chengdu, etc., have EV slow chargers available. Similarly, Europe is in second place, with over 300,000 EV slow chargers available until 2021. With 27 % of all EV slow chargers, the Netherlands is in the lead, while France has 17 %, Germany has 13 %, the United Kingdom has 10 %, Italy has 7 %, Norway has just over 4 %, and the remaining countries have 23 % each [55].

In the United States, an EV's average range and miles on a single charge are approximately 250 miles. There are three tiers of EV charging stations, each of which supplies a different maximum voltage. Level 1 has the lowest voltage and, therefore, takes the longest to charge, reflecting the corresponding increase in time [22]. Over 41,000 charging stations are available across the United States, including standard charging stations like ChargePoint and specialized options like Tesla Superchargers and Destination Chargers. Building more charging stations to accommodate the increasing number of EVs on the road has reduced the average distance between them to about 70 miles [21].

EV charging facilities are also available in small countries like Singapore. Land Transport Authority (LTA) statistics show that only about 100 charging stations are available in Singapore's commercial, residential, and public areas. The LTA has stated its intention to install 2000 charging stations across Singapore, and the ride-sharing program will also introduce one thousand new EVs into the system. However, the maximum distance between charging stations in Singapore is approximately 19.6 km [8], a much smaller area than in China, the USA, and Europe. A small country like Singapore needs to install fewer charging points for EVs. If the lower-capacity EV can travel around 60 km, installing too many charging stations would take up more space and cause traffic when charging the EVs. However, this model provides a comprehensive overview of the EV travel distance and battery capacity, which could benefit both large and small countries.

## 6 Conclusion

For EVs, it is crucial to establish guidelines that aid in distributing battery charging utilities, focusing on achieving the maximum driving ranges. The study has investigated optimal arrangements of battery power sourcing factors, which include battery capacity, charging capacity, and charging time. The authors developed an integrated model to identify the optimal energy factor orientation to achieve the minimum and maximum driving range of EVs that will reach the charging station for the next trip. The analysis results indicate that an EV with a minimum battery capacity of 10 kWh can travel up to 60–80 km. A 100-kWh battery-based EV can travel approximately 588–610 km for long distances, which depends heavily on the distance between the EV and the charging station. The model also identified that the optimum charging capacity is between 15 and 18 kW. Therefore, all types of BEVs can accommodate an average EV charging station distance of 60 km. In this framework, we studied the EV charging time, energy capacity, and battery capacity to obtain the simulated EV travel distance based on practical data analysis.

Future work will focus on expanding our framework in several different ways. One possible way is to extend the number of EV charging station installations, which could support both large and small countries. These extensions will impact vehicle models, making them more susceptible to changes in battery technology and travel distance. For instance, the average distance between charging stations in China is approximately 50 km, while the maximum distance between charging stations in Singapore is approximately 19.6 km. The alignment percentage between model-predicted data and real-time outputs showed a high precision of over 95 %. Therefore, this model could extend the overall picture to recognize the EV travel distance with battery capacity, which could be helpful for big and small countries.

## CRediT authorship contribution statement

**MD Shouquat Hossain:** Writing – review & editing, Writing – original draft, Visualization, Validation, Supervision, Software, Resources, Project administration, Methodology, Investigation, Formal analysis, Data curation, Conceptualization. **Audrius Senulis:** Writing – review & editing, Writing – original draft, Visualization, Validation, Supervision, Software, Resources, Project administration, Methodology, Investigation, Funding acquisition, Formal analysis, Conceptualization. **Laura Saltyte-Vaisiauske:** Writing – review & editing, Resources, Methodology, Funding acquisition. **Mohammad Jakir Hossain Khan:** Writing – review & editing, Writing – original draft, Visualization, Validation, Supervision, Software, Resources, Project administration, Methodology, Investigation, Formal analysis, Data curation, Conceptualization.

## Data availability

Data will be made available on request.

## Declaration of Competing Interest

The authors declare that they have no known competing financial interests or personal relationships that could have appeared to influence the work reported in this paper.
