# Supplementary material for: The optimum condition for electric vehicles’ battery powering factors to travel distance: A model-based approach
Source: Heliyon. 2024 Oct 28;10(21):e39719. doi: 10.1016/j.heliyon.2024.e39719 (PMC11582451; doi:10.1016/j.heliyon.2024.e39719)
Supplement: Multimedia component 1 [file mmc1.docx]

**Appendix A: Supplementary**

# 1. EV battery charging information

Equation 1: EV Battery charging

Controlled and uncontrolled charging formula [51]:

 (1)

where *b* is the lower and *a* is the upper limits of charging hours

For smart charging calculation, use equation (2):

 (2)

where *x_1_* is the random plug-in instant. *μ__ *and *σ__* are the average and standard deviation of *x_1_*

Battery SoC formula as a function of EV travel range [56]:

 (3)

where *R_1_* is the all-electric range of the plug-in hybrid EV in *km*

Charging duration (*D*) formula [56]:

 (4)

where *C_1_* is the battery capacity (kWh), *P* is the power rate of the charger (kW), and* * is the charger efficiency (%).

Level 2 EV charging is much faster than Level 1 in this setting. It uses an AC outlet with a voltage between 208 and 240 V in North America and 230 or 400 V in Europe [17]. In contrast, the third-stage charging voltage, or DC fast charging (DCFC), is 200–800 V. Obviously, the travel distance of EVs depends on the types of power sources. In some vehicle models, the battery lasts approximately 100 km; in others, it lasts between 200 and 400 km. The total EV driving range with a battery can be estimated using equation (5) [52]:

 (5)

where *R_total_* is the driving range, *B_e_* is the obtainable energy in batteries (kWh), and *EV_η_* is vehicle efficiency (kWh/km).

Equation (6) allows for the calculation of a battery's overall energy consumption [19]:

 (6)

where *D_R_* is the driving range per charge, *E_discharged_* is the discharged energy, *η_charge_* is the charger efficiency (%), *η_battery_* is the battery efficiency (%), and *T* is the total driving distance (km).

# 2. ANFIS model development

A fuzzy Sugeno model, the Adaptive Neural Fuzzy Inference System (ANFIS), was integrated into the framework to speed up learning and adaptation processes [57]. ANFIS aims to modify a fuzzy system's parameters by implementing a learning process using input-output training data. Like a multi-layered neural network, the structure of a neuro-fuzzy system includes both fixed and adaptive layers [58]. Fig. 1 depicts the simplified five-layer architecture of a neuro-fuzzy system with two inputs (x and y) and a single output (f). Two conditions (IF and THEN) must be met before this architecture can be considered complete.

where *x* and *y* represent inputs; *A_1_* and *B_1_* represent fuzzy sets; *f_1_* represents outputs; and *p_1_*, *q_1_*, and *r_1_* represent consequent parameters learned through training.

The first layer transforms raw data into appropriate membership functions (MF). This dynamic layer fuzzes the inputs to the membership grades of linguistic variables. Equation (7) describes the fuzzy set of inputs, which is the output *O_1_* of this layer.

 (7)

where *A(x)* and *B(y)* are membership functions of any shape, such as Gaussian or bell-shaped. *μA_i_(x)* describes the case of bell-shaped MF.

 (8)

where the MF's parameters *a_i_*, *b_i_*, and *c_i_* control the bell-shaped functions appropriately.

In the second layer, the nodes are fixed nodes. Fuzzification neurons, which stand for fuzzy sets in rule antecedents, feed information into a fuzzy rule neuron. The layer determines how important each rule is. A product of the incoming signal is used to determine this layer's output (equation (9) to (12)). The output of this layer can be represented as:

 (9)

where *w* is the weight adaptation.

The third layer is the normalized layer. In this layer, the nodes are also fixed. This layer figures out the firing strengths of each rule worked out in the previous layers. The output of this layer is computed by using equation (10).

 (10)

The fourth layer is the membership layer for the output (*Y_TD_*). The neurons in this layer demonstrate the use of fuzzy sets to apply fuzzy rules. This layer's output is the product of the normalized firing strength of the layer before it and a first-order polynomial. (11)

The defuzzification layer, the final layer, represents the neuro-fuzzy system's clear output. Equation (12) calculates this layer, which adds up each signal that enters the system.

 (12)

After the analytical exploration, we must prepare the input data described in the next section. The data will be converted to matrix-vector form so that the matrix can be used in simulation software such as MATLAB. Fig. 1 shows that the square nodes are called adaptive nodes, demonstrating that the input parameters in this node are adjustable, while the circle node is fixed to demonstrate that the parameters are fixed.


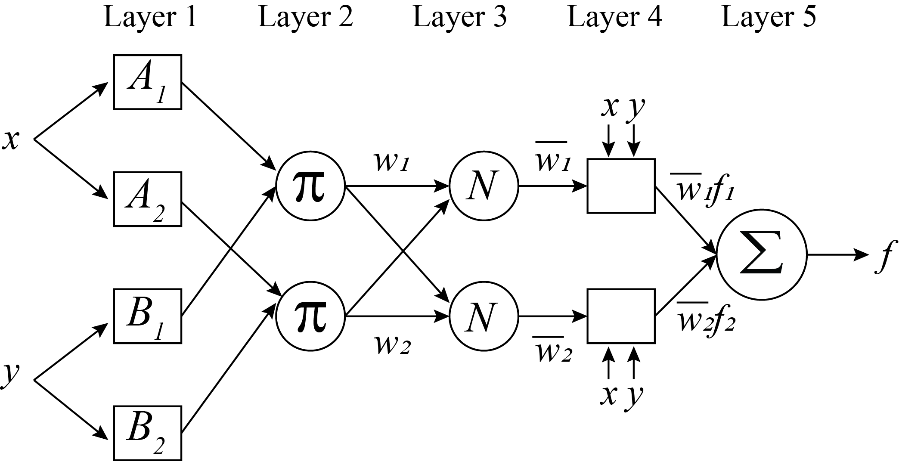


**Fig. 1.** Neuro-fuzzy architecture.

The ANFIS command was used to conduct the training process, and various training epoch sizes were evaluated while tracking the values of the root mean square error (RMSE) and mean absolute percentage error (MAPE). The formula in equations (13) and (14) was used to calculate the training errors.

 (13)

where *N* is the overall number of observations, and *yi* and *xi* are the values of the *i*^th^ observation in *y* (target value) and *x* (predicted value), respectively.

 (14)

where *At* is the actual value (target), *Ft* is the forecasted value, and *n* is the number of observations.

In this ANFIS architecture, the first and fourth layers can be seen. The first layer has three variables that can be changed: *ai*, *bi*, and *ci*. These variables are connected to the input MFs and are called premise parameters. The fourth layer has the first-order polynomial’s three variables that can be changed: *pi*, *qi*, and *ri*. These variables are called consequent parameters [59].
